# Aconitine Neurotoxicity According to Administration Methods

**DOI:** 10.3390/jcm10102149

**Published:** 2021-05-16

**Authors:** Ji Yeon Chung, Seung Jae Lee, Hyuck Jin Lee, Jeong Bin Bong, Chan-Hyuk Lee, Byoung-Soo Shin, Hyun Goo Kang

**Affiliations:** 1 Department of Neurology, Chosun University Hospital, Gwang-ju 61453, Korea; time4peace@hanmail.net (J.Y.C.); jb1986@naver.com (J.B.B.); 2 Department of Chemistry and Institute of Molecular Biology and Genetics, Jeonbuk National University, Jeonju 54907, Korea; slee026@jbnu.ac.kr; 3 Department of Chemistry Education, Kongju National University, Gongju 32588, Korea; hyuckjin@kongju.ac.kr; 4 Department of Neurology, Jeonbuk National University Medical School, Jeonju 54907, Korea; bluewave0210@gmail.com (C.-H.L.); sbsoo@jbnu.ac.kr (B.-S.S.); 5 Department of Neurology, Research Institute of Clinical Medicine of Jeonbuk National University—Biomedical Research Institute of Jeonbuk National University Hospital, Jeonju 54907, Korea

**Keywords:** aconitine, alcohol, intoxication, mechanism, neurotoxicity

## Abstract

We evaluated the toxic effects of aconitine on the human nervous system and its associated factors, and the general clinical characteristics of patients who visited the emergency room due to aconitine intoxication between 2008 and 2017. We also analyzed the differences related to aconitine processing and administration methods (oral pill, boiled in water, and alcohol-soaked), and the clinical characteristics of consciousness deterioration and neurological symptoms. Of the 41 patients who visited the hospital due to aconitine intoxication, 23 (56.1%) were female, and most were older. Aconitine was mainly used for pain control (28 patients, 68.3%) and taken as oral pills (19 patients, 46%). The patients showed a single symptom or a combination of symptoms; neurological symptoms were the most common (21 patients). All patients who took aconitine after processing with alcohol showed neurological symptoms and a higher prevalence of consciousness deterioration. Neurological symptoms occurred most frequently in patients with aconitine intoxication. Although aconitine intoxication presents with various symptoms, its prognosis may vary with the processing method and prevalence of consciousness deterioration during the early stages. Therefore, the administration method and accompanying symptoms should be comprehensively investigated in patients who have taken aconitine to facilitate prompt and effective treatment and better prognoses.

## 1. Introduction

Aconitine has been used in traditional herbal medicine to treat several chronic diseases in Asian countries for thousands of years. Aconitine is a perennial herb belonging to the Aconitum species (Ranunculaceae), which is planted globally. It is known to have anti-inflammatory, analgesic, and cardiotonic functions. It also increases systemic blood flow and improves the functioning of the digestive system [1,2,3].

**Figure 1:**
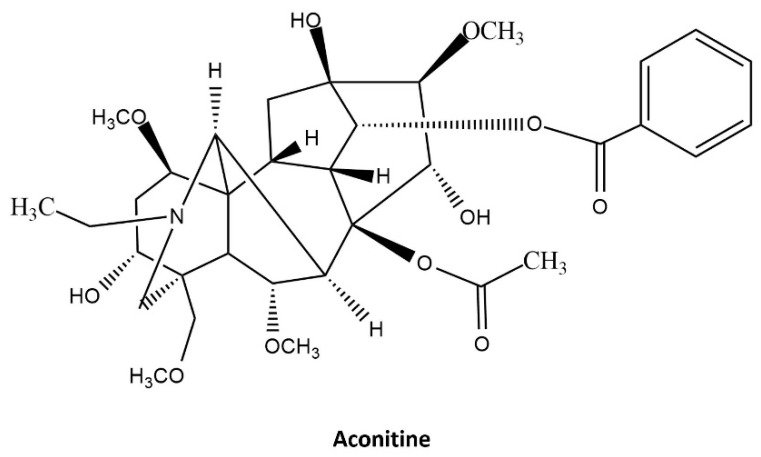
Structure of Aconitine.

Low-dose aconitine can have toxic effects because the safety margin of the toxic alkaloids in the aconitine family (Figure 1, C19-diterpenoid alkaloids: C20H32), which are found in the roots and stems of plants, is very narrow [4]. If an inadequate dosage or pretreatment is used, aconitine toxicity can cause death following gastrointestinal disorders, severe cardiac toxicity, and neurotoxicity [5,6,7]. Cardiotoxicity and neurotoxicity are the most critical effects of aconitine, and the pathological pathway of the alkaloid molecule, a crucial secondary messenger in plants, is explained using the details of the interaction of the voltage-gated sodium ion channels (VGSCs) [8,9,10]. The mechanisms underlying neurotoxicity are triggered by the interaction of aconitine with VGSCs, which enhances the state of open VGSCs [4,10,11].

There are several reports on cardiac toxicity caused by aconitine, but there are no studies on neurotoxicity associated with dosage and processing methods. We attempted to evaluate the type and frequency of neurological symptoms, their coexistence with consciousness deterioration, and their associated factors, with a focus on the effects on the nervous system (central and peripheral nervous systems).

## 2. Materials and Methods

### 2.1. Study Subjects

This study retrospectively assessed the sex, age, the purpose of taking aconitine, the processing method of aconitine, the main clinical and accompanying symptoms, underlying diseases, and vital signs of the patients obtained from their medical records. This study targeted patients who visited the emergency rooms of two university hospitals in different regions due to aconitine intoxication between January 2008 and July 2017. The two regional university hospitals were located in Jeonju and Gwangju, in the same Jeonla province, which are about 100 km (about 62 miles) apart. Aconitine intoxication was diagnosed based on the previously proposed criteria [4]: (1) experiencing abnormal symptoms immediately after taking only aconitine; (2) experiencing typical neurological, cardiogenic, and gastrointestinal symptoms after taking aconitine; (3) taking one or more medicinal herbs or aconitine roots according to the medical records.

### 2.2. Factors by Processing Method

We classified the cases into three groups based on the following processing methods to investigate the differences in toxicity: (1) processing aconitine in alcohol (i.e., soaking in alcohol, tincture made from aconite root solubilized in alcohol, and medicinal wine made from aconitine roots); (2) boiling aconitine in water (i.e., decoction, boiling aconitine in water, and steaming); (3) taking ready-made oral pills from dried extracts of aconitine. This study evaluated the influence of the processing method on changes in consciousness or clinical symptoms.

### 2.3. Factors Associated with Consciousness Deterioration

To evaluate the factors related to changes in consciousness, this study divided the subjects into clear (clear and normal consciousness) and low (confusion or consciousness deterioration) groups and compared them. The low-consciousness group also included those who developed consciousness deterioration or were unconscious, and those who showed confusion although awake.

### 2.4. Factors Associated with Neurological Symptoms

To investigate the factors related to aconitine toxicity symptoms, the patients were classified into four major groups: those with neurological, cardiogenic, gastrointestinal, and other symptoms. The neurological symptoms included dizziness, headache, tingling, deterioration of consciousness, and dysarthria, whereas the cardiogenic symptoms included hypotension and palpitations. Gastrointestinal symptoms included nausea, vomiting, abdominal pain, and diarrhea. Other symptoms included general weakness, sweating, dyspnea, salivation, urticaria/itching, and myalgia. The reason why general weakness and salivation were classified as other symptoms was that the general weakness that patients described as fatigue was classified as general weakness, and salivation can also be caused by other medical diseases. As described above, the symptoms reported by the patients were classified into neurological and non-neurological symptom groups and compared.

### 2.5. Docking Simulations

Flexible ligand docking studies on aconitine have been conducted using voltage-gated sodium channels (VGSCs; open form (PDB 5HK7) and AutoDock Vina (closed-form (PDB 3RVY)) [12,13,14]. The MMFF94 energy minimization in ChemBio3D Ultra 11.0 was used to optimize the structure of aconitine for docking studies. The structural files of aconitine and VGSC were generated by AutoDock Tools and imported into PyRx, which were used to run AutoDock Vina [14]. The search space dimensions were set to cover each VGSC (PDB: 5HK7 and 3RVY). The exhaustiveness of the docking runs was set to 1024. The docked poses of aconitine and VGSCs were visualized using PyMol software (The PyMol Molecular Graphics System, Version 2.3.4, Schrödinger, LLC, New York, NY, USA).

### 2.6. Statistical Analysis

The methods of aconitine administration were compared after classifying them into three groups: taking aconitine as oral pills from dried extracts, taking it after boiling in water, and taking it with alcohol. Consciousness deterioration was also analyzed based on demographic characteristics, and the factors associated with neurological symptoms were also analyzed. Pearson’s chi-squared test, Fisher’s exact test, Student’s *t*-test, and Wilcoxon rank-sum test were used for categorical or continuous variables, as appropriate. Univariate and multivariate analysis models were used to investigate the factors associated with low consciousness. To avoid variable selection caused by spurious correlations, only variables showing a potential association (*p* < 0.1) in the univariate analysis were included in the multivariate logistic regression model. All statistical analyses were performed using the Statistical Package for the Social Sciences version 21 (International Business Machines Corp., Armonk, NY, USA).

## 3. Results

### 3.1. Clinical and Demographic Characteristics of Patients

Forty-one patients visited two university hospitals due to aconitine intoxication within the 10 years considered in this study, and 23 of them (56.1%) were female. The proportion of older patients was higher (Supplementary Materials Figure S1). Aconitine was administered to control pain (28 patients, 68.3%) and treat indigestion (9 patients, 21.9%); some patients also mistook it for another drug (4 patients, 9.8%). Regarding the method of administration, 10 patients (24%) took it after soaking in alcohol, 12 patients (29%) took it after boiling in water, and 19 patients (46%) took it as pills. Some patients had a single symptom, while others had multiple symptoms. There were 14 patients with only single symptoms, and two symptoms appeared in one patient. One patient had up to five symptoms. Neurological symptoms were the most common (21 patients), followed by gastrointestinal (16 patients), cardiogenic (8 patients), and other (14 patients) symptoms, as described in Table 1.

### 3.2. Factors by Processing Method

The methods of aconitine administration were classified into three categories: taking it as ready-made pills from dried extracts, taking it after boiling roots in water, and taking it after processing with alcohol (soaked in alcohol).

The rate of taking pills was higher in females; more males took it after soaking in alcohol. Ten of 41 patients (24.4%) took alcohol-processed aconitine. Neurological symptoms were observed in all of the patients who took alcohol-processed aconitine. Of these ten patients, two (20%) died due to severe aconitine intoxication. Consciousness deterioration was more prevalent in those who had soaked aconitine in alcohol before taking it (Table 2).

### 3.3. Incidence of Consciousness Deterioration

The factors associated with the deterioration of consciousness were compared after dividing the patients into good- and low-consciousness groups. Of the 41 patients who took aconitine, 14 (34.1%) showed deterioration of consciousness. The mean age of the low-consciousness group was greater than that of the good-consciousness group. The incidence of consciousness deterioration was significantly higher in patients taking aconitine processed in alcohol (14.8% vs. 42.9%, *p* = 0.047). The low-consciousness group showed a higher incidence of neurological symptoms and a higher mortality rate. At the time of the visit, several patients complained of chest discomfort. Tachycardia was more prevalent in the low-consciousness group. There were no significant differences in the prevalence of hypertension and diabetes (Table 3) between the two groups. Multivariate analysis was performed on factors associated with consciousness deterioration. The results confirmed that aconitine with alcohol (odds ratio (OR) 11.76 (1.86–74.11), *p* = 0.009) was significantly associated with low consciousness, and chest discomfort was associated with clear consciousness (OR 0.14 (1.51–50.86), *p* = 0.002, Table 3).

### 3.4. Factors Associated with Neurological Symptoms

Neurological symptoms were observed in 21 (51.2%) of 41 patients. Age, mortality, cardiovascular symptoms, gastrointestinal symptoms, and the presence of underlying diseases were investigated for their possible associations with the occurrence of neurological symptoms; they did not differ significantly in the groups of patients with and without neurological symptoms. Interestingly, all ten patients who took aconitine after processing with alcohol showed neurological symptoms (Table 4).

## 4. Discussion

This study investigated the purpose and administration methods of aconitine and the clinical symptoms of patients who visited the emergency room due to intoxication. The study also evaluated factors associated with neurological symptoms. The most common reason for taking aconitine is to relieve pain, and taking it as oral pills is the most common administration method. Neurological symptoms were most frequently observed in this study, followed by gastrointestinal and cardiogenic symptoms. All patients who took aconitine after processing with alcohol (soaked in alcohol) showed neurological symptoms and a significantly higher prevalence of consciousness deterioration and neurological symptoms than those who took it in other ways.

Since there is no special regulation for the preparation of aconitine, intoxication occasionally occurs. C19, a deadly poisonous alkaloid extracted from aconitine, is highly toxic when used as herbal medicine [1,10,15]. Since the quantity and composition of alkaloids vary with the harvesting time, variety, and processing method, the degree of intoxication changes. Therefore, physiochemical manipulations through appropriate pretreatment, and processing to reduce toxicity or side effects are important to ensure safety. Ordinary people without specialized knowledge use what is collected from the mountains and fields directly and what is purchased from the medicine market without proper processing, and they frequently experience intoxication and side effects. If there are no pharmaceutical grades or standards for particular substances, the number of active ingredients in dietary supplements will vary greatly. Since there is no standardized processing method, and manufacturers produce aconitine pills based on their empirical methods, the content of aconitine in a pill has not been established. Therefore, there are no standard protocols for assessing drug quality. The sales and preparation of aconitine are legally regulated in China and Taiwan. It is believed that other countries using aconitine, including South Korea, will need to regulate the preparation of aconitine as well.

Previous studies have shown that people generally take aconitine to control pain [1,16], and this study also revealed that most subjects used aconitine for pain control. The modalities of administration were classified into three main categories: taking it after soaking in alcohol, taking it after boiling in water, and taking it as a manufactured pill. Taking a pill was the most common modality of administration (46.3%). It is believed that pills were used the most because they were easy to obtain from the traditional market or oriental pharmacy. Aconitine intoxication results in various systemic symptoms depending on the dose and individual sensitivity. Symptoms can be divided into neurological, cardiogenic, and gastrointestinal symptoms. Several cardiogenic symptoms, such as palpitations, have been reported, and Chan (2012) showed that neurological symptoms, such as systemic paresthesia, were the most common [1]. As reported by Chan et al. (1994), neurological symptoms were the most prevalent in this study, followed by gastrointestinal and cardiogenic symptoms [8]. Chan (2009) also indicated that nausea and vomiting were the most common early symptoms, and a burning sensation around the mouth, abnormal sensation, diarrhea, abdominal pain, and chest pain occurred in isolation or combination [17]. In this study, a tingling of the limbs, accompanied by nausea, vomiting, and dizziness as initial symptoms was frequent, and a single symptom or a combination of symptoms was observed. Our findings were similar to those reported in other studies.

Consciousness deterioration was observed in 14 patients (34.1%); it was frequently observed in older adults and those who took alcohol-processed aconitine. Moreover, the group with the deterioration of consciousness showed a higher prevalence of neurological symptoms, chest discomfort, and tachycardia, and a higher mortality rate. The results indicated that when aconitine was processed with alcohol, the level of toxicity, the incidence of neurological symptoms and low consciousness, and the mortality rate increased. Aconitine comprises a nitrogen atom attached to one of the six-membered rings (Figure 1). When boiled in water for 1.5–2 h, the aconitine alkaloid is transformed into a benzylaconitine derivative. Since boiling decreases toxicity by approximately 10%, it has been recommended that aconitine should be taken after decocting in water to ensure medicinal safety [10]. Aconitine is dissolved in alcohol more efficiently. Therefore, soaking in alcohol increases the concentration of toxic substances more than the other processing methods. Consequently, the severity of intoxication changes. Due to this difference in solubility, all patients who took aconitine soaked in alcohol showed more neurological symptoms, and the prevalence of neurological symptoms was significantly higher than that of cardiogenic or gastrointestinal symptoms.

Aconitine maintains the depolarization state by binding to the neurotoxin receptor binding site II located in the alpha subunit of the transmembrane region in the voltage-dependent sodium channel of the nerve, muscle, and cardiac muscle cells. This mechanism reduces neuromuscular transmission by stimulating various cell membranes in sensory and motor nerve axons. At therapeutic concentrations, this mechanism inhibits pain conduction and has beneficial effects [5,18]. In addition to the purpose of pain control or digestion, there are reports that aconitine has been tried for the purpose of alleviating other neurological diseases such as depression, epilepsy, and dementia. The mechanism aimed to increase the expression of BDNF or to control the sensitivity to serotonin [19].

Aconitine can induce neurological symptoms, such as numbness and tingling in the mouth and limbs, and general weakness; it can also affect the cardiogenic system at toxic concentrations. We could not objectively determine the exact quantity of aconitine taken by each subject in this study. It was difficult to accurately calculate the amount taken, but neurological symptoms were confirmed in all the patients who took aconitine after soaking in alcohol, whose occurrence is thought to be more frequent because aconitine dissolves better in alcohol. Although it has not yet been established, this may occur because the decrease in sensory and motor neurons due to lowered neuromuscular transmission affects the neurological system more quickly than the cardiogenic system during the early stages with a lower dose. Moreover, it is believed that a lower dose of aconitine is required to induce neurological rather than cardiogenic symptoms. The results of this study showed that the toxicity of aconitine increased when processed with alcohol, as reported in previous studies [20]. The risk of neurological symptoms increases with alcohol consumption.

**Figure 2:**
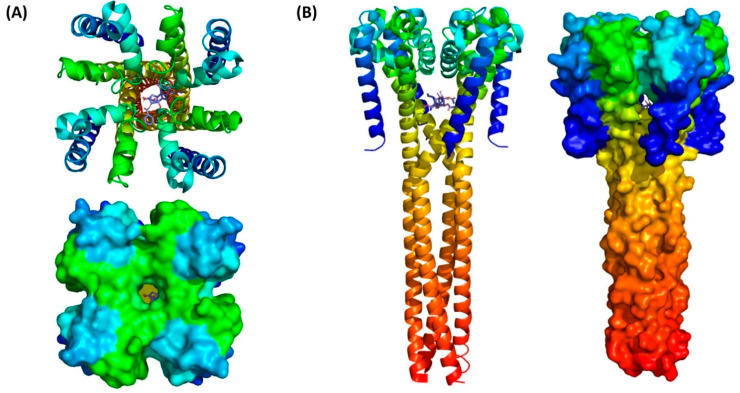
Molecular dynamic simulation results show that aconitine is positioned inside the channel below the four-helix bundle through hydrophobic and hydrogen bonds. (**A**) Top and (**B**) side views of aconitine with voltage-gated sodium ion channels (PDB: 3RVY) illustrated with ribbon diagrams and surface models.

The biochemical properties of aconitine, a C19-norditerpenoid alkaloid, may account for its physiological effects (Figure 2a). It has more than 15 hydrogen-bond donors and acceptors, but low water solubility. In this study, the solubility of aconitine significantly improves with the addition of alcohol or organic solvents, and absorption and toxicity can be monitored based on these solubility issues. Structural studies on VGSCs are difficult because these integral membrane proteins change their conformation through diverse signaling pathways. In addition, their sizes are larger than 200 kDa, with 24 transmembrane domains that enhance their complexity. To overcome these difficulties, bacterial sodium channels are used to investigate the mechanisms by which the prokaryotic system replaces the mammalian sodium channel with a simpler structure.

To visualize the potential interactions of aconitine with voltage-gated sodium channels (VGSCs; open-form (PDB 5HK7) and closed-form (PDB 3RVY)), flexible ligand docking studies were conducted using AutoDock Vina [12,13,14]. Most of the docked conformations of aconitine with open-form VGSCs were found at the entrance or within the channel, and they could inhibit the transport of sodium ions (Figure 2a). Aconitine also interacted with closed-form VGSCs on the sides of the channels (Figure 2b).

Among the symptoms observed in this study, the mechanisms underlying hypotension, palpitations, and dyspnea related to the effects of aconitine toxicity on the central nervous system can be evaluated. A previous study directly injected aconitine into the peritonea of rats and observed that the amount of acetylcholine released increased in the frontal lobe, regardless of the stimulation of the muscarinic receptor in the central nervous system [18]. Through this mechanism, the spinal cord was stimulated to lower the heart rate and blood pressure. If a similar mechanism influences the central nervous system in the human body, the same symptoms may occur. Another possibility is that aconitine may lead to hypotension by activating muscarinic receptors [21]. It can directly affect myocardial contraction by inhibiting myocardial repolarization. Aconitine can also cause calcium overload through the exchange of sodium and calcium or the opening of the sodium passage, which may also be caused by promoting the triggering activity of the myocardium, increasing parasympathetic activity, and decreasing atrioventricular conduction [11]. Consciousness deterioration may be caused by cerebral hypoperfusion due to hypotension. It is a basic hypothesis that aconitine affects theVGSC of the central nervous system and causes loss of consciousness, but it may be also capable of inducing a decrease in consciousness by causing cardiac toxicity, leading to a decrease in cardiac output, and then to a decrease in blood flow to the brain.

This study has several limitations. First, aconitine intoxication was determined based only on the medical records of the patients, since this was a retrospective study. The symptoms developed after taking aconitine, and the time taken to reach the hospital varied. Therefore, symptoms that disappeared before arrival in the emergency room were not known. Second, we evaluated the administration patterns of aconitine. The results showed that the subjects took aconitine in various ways. As a result, we could not exclude the possibility that ingredients other than aconitine caused toxic effects. Moreover, it was impossible to determine the content of aconitine in the aconitine-soaked alcohol or pills the subjects took, the types of excipients constituting the pills, and the exact content of aconitine contained in the raw root. Furthermore, the same dose can cause different types and severities of symptoms in different people because of differences in personal sensitivity to physiological functions. These two limitations could be compensated for by measuring the blood or urinary aconitine concentration at the time of admission, and examining the patient’s neurological symptoms and their severity, as well as comparable interrelated factors.

## 5. Conclusions

This study compared and analyzed various symptoms and administration methods for patients who developed intoxication symptoms after taking aconitine. These results indicate that aconitine intoxication can manifest through various symptoms, and prognosis may vary depending on the administration method and consciousness deterioration during the early stages. This may lead to fatal consequences. If a patient visits a hospital due to aconitine intoxication, it will be necessary to identify the administration method and evaluate the accompanying symptoms through a detailed medical history to understand and predict the progression of patient symptoms and prognosis. It will also be necessary to increase public awareness of the dangers of aconitine.

## Figures and Tables

**Table 1:** Baseline characteristics of clinical symptoms.

Correlated Symptoms	Number of Patients (*n* = 41)
Nausea/vomiting	18 (43.9)
Dizziness	10 (24.4)
Tingling sense	8 (19.5)
Chest discomfort	7 (17.0)
General weakness	6 (14.6)
Mental deterioration	6 (14.6)
Hypotension	4 (10.0)
Abdominal pain	4 (10.0)
Diarrhea	3 (7.3)
Sweating	3 (7.3)
Palpitation	2 (4.9)
Dysarthria	2 (4.9)
Dyspnea	2 (4.9)
Salivation	1 (2.4)
Headache	1 (2.4)
Urticaria/itching sense	1 (2.4)
Myalgia	1 (2.4)

**Table 2:** General characteristics of processing methods.

	**Pills** **(*n* = 19)**	**Boiled Water** **(*n* = 12)**	**with Alcohol** **(*n* = 10)**	***p*-Value**
Female	14 (73.7)	6 (50.0)	3 (30.0)	0.07
Age	65.53 ± 19.65	63.92 ± 11.14	63.70 ± 10.62	0.941
Death	0 (0)	0 (0)	2 (20.0)	0.038
Neurological symptoms	6 (31.6)	5 (41.7)	10 (100)	0.003
Cardiological symptoms	2 (10.5)	5 (41.7)	1 (10.0)	0.071
Gastric symptoms	6 (31.6)	5 (41.7)	5 (50.0)	0.611
Mental deterioration	5 (26.3)	3 (25.0)	6 (60.0)	0.14
Taking purpose (for pain control)	13 (68.4)	10 (83.3)	5 (50.0)	0.247
Pre-existing factor				
Hypertension	8 (44.4)	6 (50.0)	4 (40.0)	0.894
Diabetes	3 (16.7)	2 (16.7)	2 (20.0)	0.972
Chest discomfort	4 (21.1)	4 (33.3)	1 (10.0)	0.417
Heart rate at visit	73.79 ± 16.02	96.83 ± 41.51	95.60 ± 45.28	0.111
	**without Alcohol** **(*n* = 31)**		**with Alcohol** **(*n* = 10)**	***p*-Value**
Female	20 (64.5)		3 (30.0)	0.056
Age	64.90 ± 16.67		63.70 ± 10.62	0.832
Death	0 (0)		2 (20.0)	0.055
Neurological symptoms	11 (35.5)		10 (100)	<0.001
Cardiological symptoms	7 (22.6)		1 (10.0)	0.383
Gastric symptoms	11 (35.5)		5 (50.0)	0.413
Mental deterioration	8 (25.8)		6 (60.0)	0.047
Taking purpose (for pain control)	23 (74.2)		5 (50.0)	0.153
Hypertension	14 (46.7)		4 (40.0)	0.714
Diabetes	5 (16.7)		2 (20.0)	0.81
Chest discomfort	8 (25.8)		1 (10.0)	0.294
Heart rate at visit	82.71 ± 30.27		95.60 ± 45.28	0.308

**Table 3:** Factors associated with consciousness deterioration.

	**Clear Consciousness (*n* = 27)**			**Low Consciousness (*n* = 14)**		***p*-Value**
Female	16 (59.3)			7 (50.0)		0.571
Age	61.00 ± 16.25			71.57 ± 10.63		0.034
Death	0 (0)			2 (14.3)		0.044
Neurological symptoms	7 (25.9)			14 (100)		<0.001
Cardiological symptoms	3 (11.1)			5 (35.7)		0.059
Gastric symptoms	12 (44.4)			4 (28.6)		0.323
Taking purpose (for pain control)	20 (74.1)			8 (57.1)		0.269
Taking method (with alcohol)	4 (14.8)			6 (42.9)		0.047
Hypertension	10 (37.0)			8 (61.5)		0.145
Diabetes	6 (22.2)			1 (7.7)		0.393
Chest discomfort	3 (11.1)			6 (42.9)		0.02
Heart rate at visit	76.33 ± 21.59			104.21 ± 46.32		0.049
	**Crude OR (95% CI)**		***p*-Value**	**Adjusted OR (95% CI)**		***p*-Value**
Chest discomfort	0.34	0.96–19.31	0.01	0.14	1.52–50.86	0.002
Taking method (with alcohol)	6.00	1.21–29.72	0.28	11.76	1.87–74.11	0.009

Results are expressed as odds ratio and 95% confidence interval. Variables with *p* < 0.1 by univariate analysis were entered into the multivariate analysis model. CI, confidence interval; OR, odds ratio.

**Table 4:** Factors associated with neurological symptoms.

	Patients without Neurological Symptoms (*n* = 20)	Patients with Neurological Symptoms (*n* = 21)	*p*-Value
Female	13 (65.0)	10 (47.6)	0.262
Age	61.55 ± 18.06	67.52 ± 11.82	0.216
Death	0 (0)	2 (9.5)	0.488
Cardiological symptoms	3 (15.0)	5 (23.8)	0.697
Gastric symptoms	8 (40.0)	8 (38.1)	0.901
Taking purpose (for pain control)	15 (75.0)	13 (61.9)	0.368
Taking method (with alcohol)	0 (0)	10 (47.6)	<0.001
Hypertension	9 (45.0)	9 (45.0)	1
Diabetes	3 (15.0)	4 (20.0)	1
Chest discomfort	2 (10.0)	7 (33.3)	0.13
Heart rate at visit	76.00 ± 19.82	95.24 ± 42.39	0.071

## Data Availability

Anonymized data, statistical methods, and experimental material not entirely published within the article will be shared by request from any qualified investigator.

## Supplementary Materials

The following are available online at https://www.mdpi.com/article/10.3390/jcm10102149/s1, Figure S1: Distribution of patients according to age.
