# Supplementary figures and images for: Aconitine Neurotoxicity According to Administration Methods
Source: J Clin Med. 2021 May 16;10(10):2149. doi: 10.3390/jcm10102149 (PMC8155921; doi:10.3390/jcm10102149)

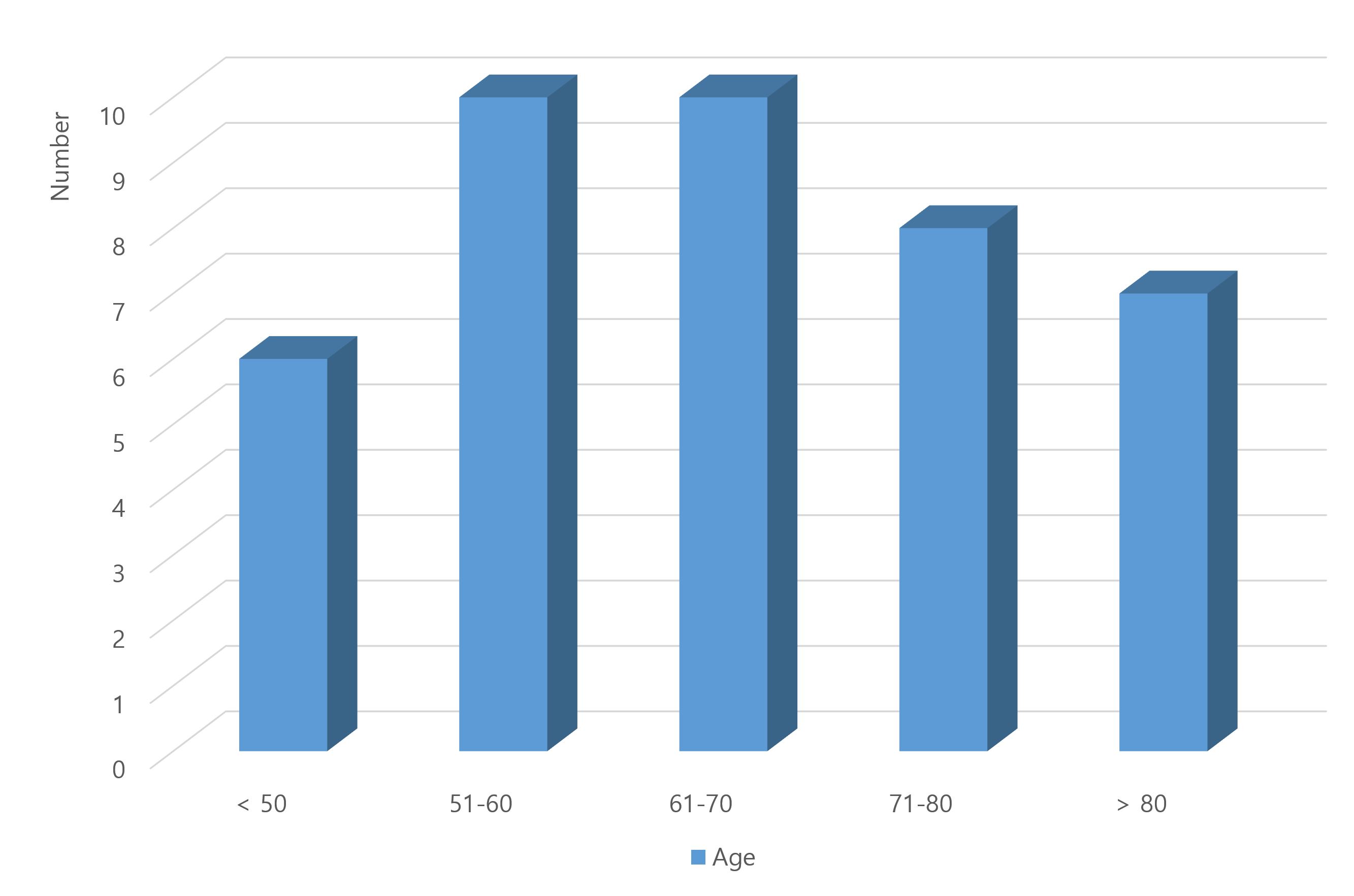

Supplement: Supplementary file 1 [file jcm-10-02149-s001.zip › Suppl Fig.JPG]
